# Assessing the performance of ChatGPT and Bard/Gemini against radiologists for Prostate Imaging-Reporting and Data System classification based on prostate multiparametric MRI text reports

**DOI:** 10.1093/bjr/tqae236

**Published:** 2024-11-13

**Authors:** Kang-Lung Lee, Dimitri A Kessler, Iztok Caglic, Yi-Hsin Kuo, Nadeem Shaida, Tristan Barrett

**Affiliations:** Department of Radiology, University of Cambridge, Cambridge CB2 0QQ, United Kingdom; Department of Radiology, Cambridge University Hospitals NHS Foundation Trust Addenbrooke’s Hospital, Cambridge CB2 0QQ, United Kingdom; Department of Radiology, Taipei Veterans General Hospital, Taipei 112, Taiwan; School of Medicine, National Yang Ming Chiao Tung University, Taipei 112, Taiwan; Department of Radiology, University of Cambridge, Cambridge CB2 0QQ, United Kingdom; Department of Radiology, Cambridge University Hospitals NHS Foundation Trust Addenbrooke’s Hospital, Cambridge CB2 0QQ, United Kingdom; Department of Radiology, Cambridge University Hospitals NHS Foundation Trust Addenbrooke’s Hospital, Cambridge CB2 0QQ, United Kingdom; Department of Radiology, Taipei Veterans General Hospital, Taipei 112, Taiwan; Department of Radiology, Cambridge University Hospitals NHS Foundation Trust Addenbrooke’s Hospital, Cambridge CB2 0QQ, United Kingdom; Department of Radiology, University of Cambridge, Cambridge CB2 0QQ, United Kingdom; Department of Radiology, Cambridge University Hospitals NHS Foundation Trust Addenbrooke’s Hospital, Cambridge CB2 0QQ, United Kingdom

**Keywords:** prostate MRI, PI-RADS, large language model, ChatGPT, Bard, Gemini

## Abstract

**Objectives:**

Large language models (LLMs) have shown potential for clinical applications. This study assesses their ability to assign Prostate Imaging-Reporting and Data System (PI-RADS) categories based on clinical text reports.

**Methods:**

One hundred consecutive biopsy-naïve patients’ multiparametric prostate MRI reports were independently classified by 2 uroradiologists, ChatGPT-3.5 (GPT-3.5), ChatGPT-4o mini (GPT-4), Bard, and Gemini. Original report classifications were considered definitive.

**Results:**

Out of 100 MRIs, 52 were originally reported as PI-RADS 1-2, 9 PI-RADS 3, 19 PI-RADS 4, and 20 PI-RADS 5. Radiologists demonstrated 95% and 90% accuracy, while GPT-3.5 and Bard both achieved 67%. Accuracy of the updated versions of LLMs increased to 83% (GTP-4) and 79% (Gemini), respectively. In low suspicion studies (PI-RADS 1-2), Bard and Gemini (F1: 0.94, 0.98, respectively) outperformed GPT-3.5 and GTP-4 (F1:0.77, 0.94, respectively), whereas for high probability MRIs (PI-RADS 4-5), GPT-3.5 and GTP-4 (F1: 0.95, 0.98, respectively) outperformed Bard and Gemini (F1: 0.71, 0.87, respectively). Bard assigned a non-existent PI-RADS 6 “hallucination” for 2 patients. Inter-reader agreements (*Κ*) between the original reports and the senior radiologist, junior radiologist, GPT-3.5, GTP-4, BARD, and Gemini were 0.93, 0.84, 0.65, 0.86, 0.57, and 0.81, respectively.

**Conclusions:**

Radiologists demonstrated high accuracy in PI-RADS classification based on text reports, while GPT-3.5 and Bard exhibited poor performance. GTP-4 and Gemini demonstrated improved performance compared to their predecessors.

**Advances in knowledge:**

This study highlights the limitations of LLMs in accurately classifying PI-RADS categories from clinical text reports. While the performance of LLMs has improved with newer versions, caution is warranted before integrating such technologies into clinical practice.

## Introduction

Prostate cancer (PCa) is the second commonest male cancer globally,^1^ with MRI being the primary diagnostic tool for detection.^2^ The Prostate Imaging-Reporting and Data System (PI-RADS) guidelines aim to standardize MRI reporting and have been widely adopted in the uroradiology community.^2^ Radiologists typically analyse images, formulate descriptors, and synthesize these into impressions, including the designation of a PI-RADS score, with this categorization used to guide clinicians in management decisions.^2^^,^^3^ Previous literature has demonstrated high inter-observer variability in PI-RADS classification among radiologists^4^; however, these studies have focused on the entire process of designating a PI-RADS category.

Large language models (LLMs), such as Chat Generative Pre-trained Transformer (ChatGPT) from OpenAI and Bard/Gemini developed by Google, have attracted attention for their potential applications in many disciplines, including clinical radiology.^5^^,^^6^ Trained on extensive web text data, these LLMs exhibit the capability to comprehend and generate responses across diverse contexts in human language, with the potential to translate clinical radiology reports into patient-friendly lay text.^7^ The initial stage in this process is establishing a reliable clinical interpretation capability. Hence, evaluating the ability of LLMs to generate a single PI-RADS classification from a text-based report is of interest. This study aims to compare the classification abilities of ChatGPT, Bard/Gemini, and 2 uroradiologists in assigning PI-RADS categories based on the initial clinical text reports.

## Methods

### Original radiology reports

**Figure 1.**
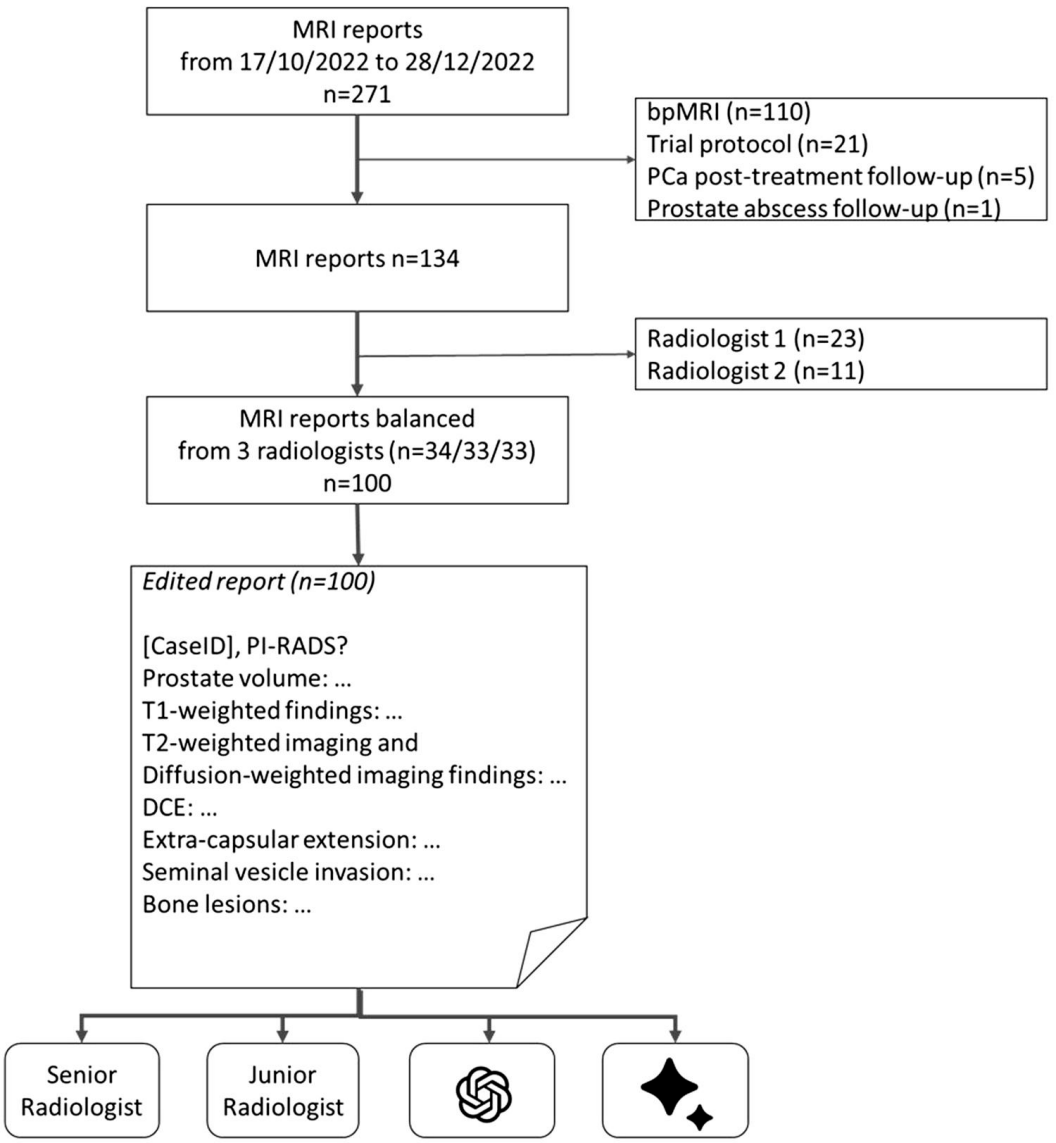
The flow diagram illustrates the study’s text report inclusion process and study pipeline. The study involved the inclusion of reports from clinical routine multiparametric MRI scans of treatment-naïve patients, ensuring a balanced distribution with 3 radiologists (*n* = 34, 33, 33). These reports underwent editing through the addition of a structured prompt, “[CaseID], PI-RADS?”, positioned at the start of each report, and the removal of clinical history, concluding remarks, and PI-RADS classification. Following these revisions, the reports were evaluated by 2 radiologists, ChatGPT, and Bard/Gemini. Abbreviations: bpMRI = biparametric MRI; DCE = dynamic contrast enhanced images; PCa = prostate cancer; PI-RADS = Prostate Imaging-Reporting and Data System.

This retrospective analysis was approved by the institutional review board, with the need for informed consent waived (*anonymized*). The study was conducted on clinical prostate MRI text reports derived from a cohort of 100 consecutive biopsy-naïve men who underwent multiparametric MRI, with a balanced distribution across 3 reporting radiologists, between October 17, 2022 and December 28, 2022 (Figure 1). The radiologists were considered experts,^8^^,^^9^ and contributed 34, 33, and 33 reports, respectively.

### Reports editing, PI-RADS classification and prompting

In our institution, prostate MRIs are reported in a structured format. To ensure a standardized blinded assessment, the clinical history, concluding remarks, and PI-RADS classification were removed from the text reports. Two uroradiologists (*anonymized*), with 14 and 3 years of prostate MRI reporting experience, independently and retrospectively classified PI-RADS 2.1 categories from the edited text reports.

The same reports were manually inputted into the online ChatGPT-3.5 (GPT-3.5) and Google Bard (Bard) platforms to perform zero-shot generation of PI-RADS 2.1 categories without prior training between September 18, 2023 to October 08, 2023. To assess the performance of the updated ChatGPT-4o mini (GTP-4) and Google Gemini-1.5 (Gemini) models, the same procedure (identical prompt and zero-shot training) was repeated on the online chatbots between August 27, 2024 and September 06, 2024.

For the purposes of repeatability analysis, the 2 uroradiologists classified PI-RADS categories from the same reports with a washout period more than 9 months between evaluations. Each case evaluated 3 queries submitted via the Application Programming Interfaces (APIs) of GTP-4 and Gemini. Python version 3.9.12 (Python Software Foundation, Wilmington, DE, United States) was used for the implementation, leveraging the openai package (version 1.6.1) for GPT API interactions, and the google.generativeai package (version 0.8.0) for Google Gemini API queries. The model version used for ChatGPT was gpt-4o-mini-2024-07-18, while the Gemini model was gemini-1.5-flash-001.

The prompts for both ChatGPT and Google BARD/Gemini were structured as “[CaseID], PI-RADS?”, with the [CaseID] representing a unique identifier for each individual report (Figure 1).

### Statistical analysis

The original report classifications were considered as definitive reference standards. Subsequent analyses focused on comparing these original reports with the assessments made by the radiologists, ChatGPT, and Bard. In the original classifications, PI-RADS 1 and 2 were collectively grouped due to their absence of any distinct clinical meaning. Consequently, PI-RADS 1 and 2 were amalgamated into a single classification for analysis. To assess whether there were significant differences in the assignment of PI-RADS, Fisher’s exact test or Chi-squared test were used where appropriate, and *P* < .05 was considered significant. Performance of the raters was evaluated in terms of accuracy and F1-scores. The F1 scores were determined by the harmonic mean of macro averaged precision and macro averaged recall in relation to the definitive classifications provided in the original reports. The clinical decision to perform a prostate biopsy is strongly guided by PI-RADS scores, commonly based on grouped categories of PI-RADS 1-2 (low suspicious of PCa), 3 (indeterminate), and 4-5 (high probability of PCa).^10^ Consequently, Kappa analyses and subgroup performance analyses were performed on these grouped categories with inter-reader reliability (*Κ*): <0.00, poor agreement; 0.00-0.20, slight; 0.21-0.40, fair; 0.41-0.60, moderate; 0.61-0.80, substantial; and 0.81-1.00, almost perfect.^11^ The repeatability of GTP-4 and Gemini was evaluated by comparing the PI-RADS classifications generated by the web-based chatbots with the results from 3 iterations using the respective LLM APIs. The repeatability was determined by calculating the proportion of cases where the same PI-RADS score was given across all attempts for each model. A percentage agreement rate of <90% for the identical answer choices across attempts was classified as poor.^12^ R 4.2.2 (R Foundation, Vienna, Austria) and Python 3.9.13 were used for statistical analysis.

## Results

In the cohort of 100 original clinical prostate MRI reports, 52 MRIs were categorized as PI-RADS 1-2, 9 as PI-RADS 3, 19 as PI-RADS 4, and 20 as PI-RADS 5 (Table 1). The distribution of PI-RADS categories was not significantly different across the 3 radiologists who made the original reports (*P* = 0.41, Table S1).

**Table 1.** PI-RADS scores breakdown across original text reports, 2 human raters (SR, JR), and pre-trained large language models (GPT, Bard, Gemini).

PI-RADS	Original	SR	JR	GPT-3.5	GTP-4	Bard	Gemini
1-2	52	54	51	34	48	55	54
3	9	9	8	29	11	21	14
4	19	17	20	23	30	10	22
5	20	20	21	14	11	12	10
6	0	0	0	0	0	2	0

Abbreviations: GPT-3.5 = ChatGPT-3.5; GTP-4 = ChatGPT-4o mini; JR = Junior Reader; PI-RADS = Prostate Imaging-Reporting and Data System; SR = Senior Reader.

**Figure 2.**
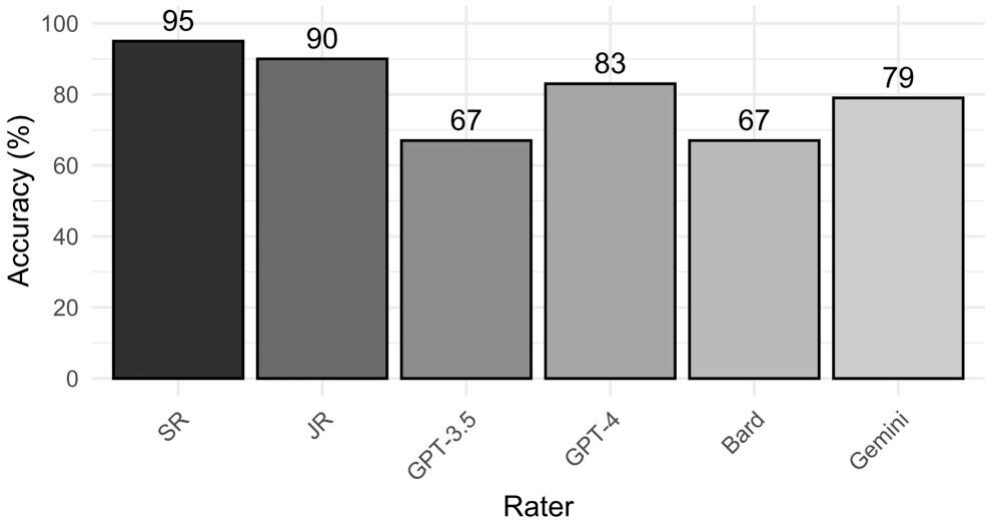
Accuracy of PI-RADS categorization compared to the categories in the original prostate MRI reports across raters. Abbreviations: GPT-3.5 = ChatGPT-3.5; GTP-4 = ChatGPT-4o mini; JR = Junior Reader; PI-RADS = Prostate Imaging-Reporting and Data System; SR = Senior Reader.

When comparing original classifications to assessments made by the senior radiologist (SR) and junior radiologist (JR), high levels of accuracy were observed, with the SR achieving 95% and the JR achieving 90% accuracy. GPT-3.5 and Bard both achieved a lower overall accuracy of 67% (Table 2, Figure 2). However, the accuracy of GTP-4 (83%) showed a significant improvement compared to GPT-3.5 (*P* = .009). While Gemini’s accuracy (79%) also improved over Bard, this difference was not statistically significant (*P* = .056). Additionally, both radiologists exhibited high macro F1 scores, with the SR achieving 0.91 and the JR 0.82, with both outperforming GPT-3.5 (0.64), GTP-4 (0.76), Bard (0.41), and Gemini (0.68), respectively. There was no distinct pattern indicating that LLMs’ performance differed across the 3 original radiologists (Table S2).

**Table 2.** Performance for PI-RADS classification across 2 human raters (SR, JR), and the pre-trained large language models (GPT, Bard/Gemini).

	SR	JR	GPT-3.5	GTP-4	Bard	Gemini
PI-RADS 1-2 F1 Score	0.98	0.95	0.77	0.94	0.94	0.98
PI-RADS 3 F1 Score	0.78	0.47	0.42	0.60	0.20	0.52
PI-RADS 4-5 F1 Score	0.97	0.95	0.95	0.98	0.71	0.87
All PI-RADS						
Accuracy	0.95 (95/100)	0.90 (90/100)	0.67 (67/100)	0.83 (83/100)	0.67 (67/100)	0.79 (79/100)
Macro F1 Score	0.91	0.82	0.64	0.76	0.41	0.68
K	0.93	0.84	0.65	0.86	0.57	0.81

Abbreviations: GPT-3.5 = ChatGPT-3.5; GTP-4 = ChatGPT-4o mini; JR = Junior Reader; K = inter-reader agreement (Kappa) between the original report and the listed readers; PI-RADS = Prostate Imaging-Reporting and Data System; SR = Senior Reader.

**Figure 3.**
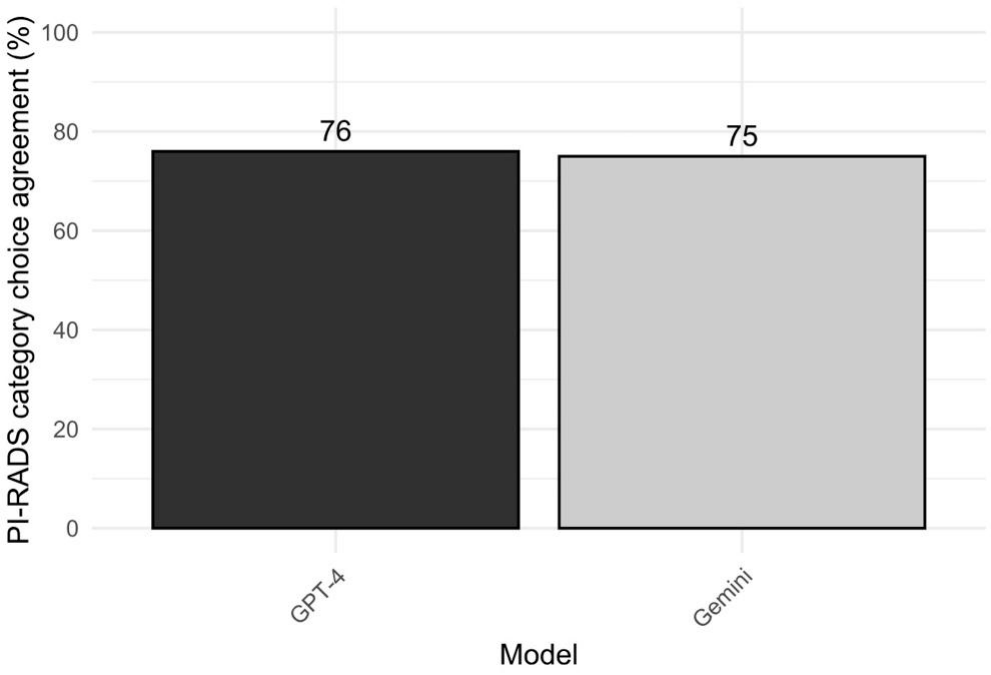
Repeatability of PI-RADS category choice agreement within ChatGPT-4o mini (GTP-4) and Gemini. Abbreviation: PI-RADS = Prostate Imaging-Reporting and Data System.

The assessment of inter-reader agreement revealed almost perfect agreement between original reports and the SR (*Κ* = 0.93) and JR (*Κ* = 0.84). Conversely, the agreement levels between the original reports and GPT-3.5 and Bard were substantial (*Κ* = 0.65) and moderate (*Κ* = 0.57), respectively. Agreement levels compared to original reports upgraded to almost perfect for both GTP-4 (*Κ* = 0.86) and Gemini (*K* = 0.81), respectively. The repeatability of SR (92%) and JR (94%) was reliable with an almost perfect intra-reader agreement for each of the readers (SR, *K* = 0.87; JR, *K* = 0.93). However, the repeatability of GTP-4 was only 76%, and Gemini 75%, indicating a lower level of repeatability for both models (Figure 3). Intra-reader agreement was almost perfect for GTP-4 (*K* = 0.86) and substantial for Gemini (*K* = 0.79).

**Figure 4.**
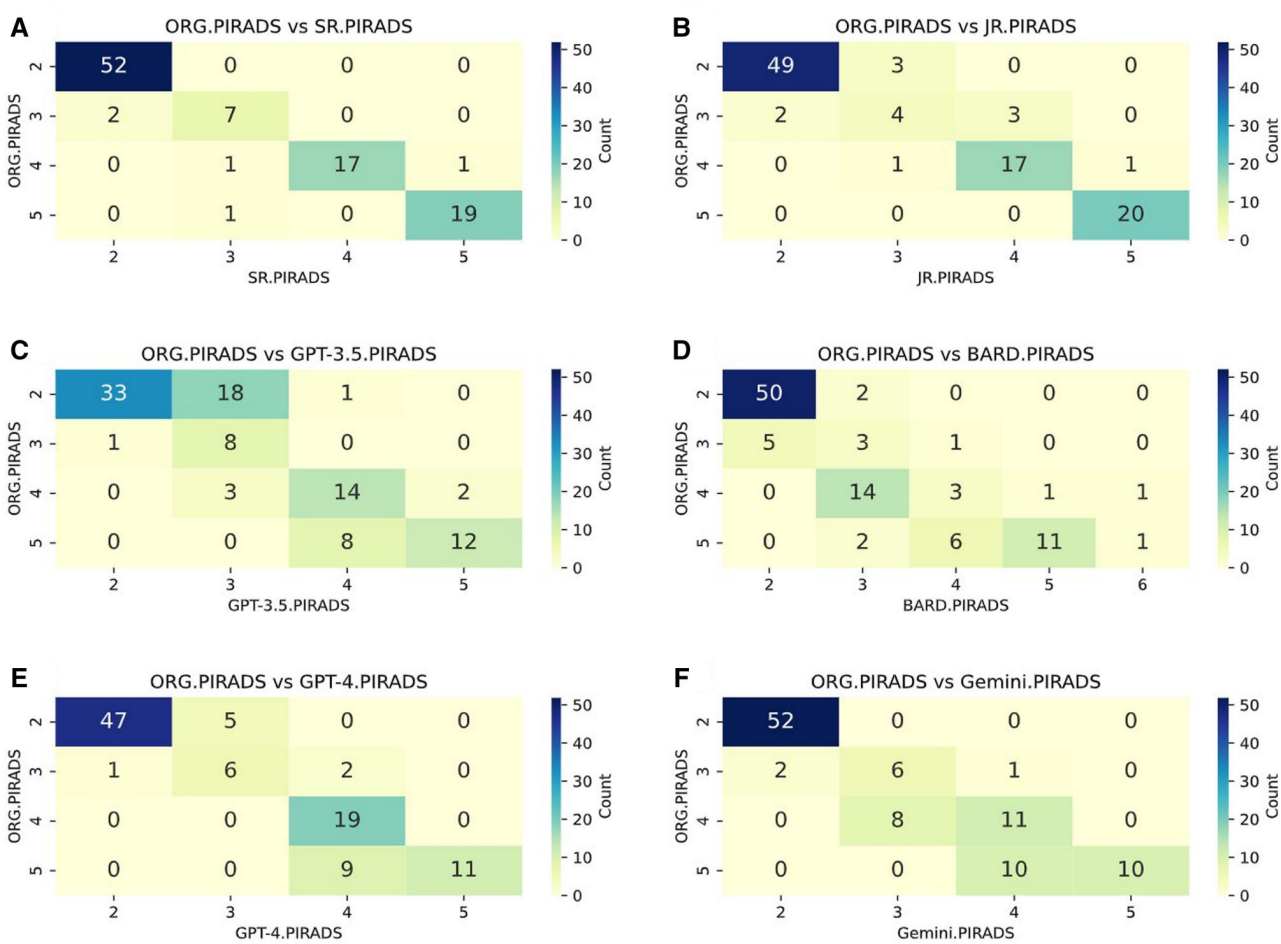
Heatmaps illustrating the distribution of PI-RADS scores. Heatmap showing the distribution of PI-RADS categories of the original text reports across PI-RADS categories of (A) senior radiologist, (B) junior radiologist, (C) GPT-3.5, (D) Bard, (E) GTP-4, (F) Gemini, respectively. In each heatmap, colours represent the count of cases corresponding to specific PI-RADS scores. The colour bar on the side of each heatmap indicates the count range, with darker shades representing higher counts. Abbreviations: GPT-3.5 = ChatGPT-3.5; GTP-4 = ChatGPT-4o mini; JR = Junior Reader; ORG = original; PI-RADS = Prostate Imaging-Reporting and Data System; SR = Senior Reader.

Within the subset of original reports designated as PI-RADS 1-2 (*n* = 52), the SR achieved an F1 score of 0.98 and the JR 0.95 (Figure 4, Table 2). Notably, none of the radiologists, BARD, nor Gemini categorized any report within this subset as PI-RADS 4 or 5, with F1 scores ranging from 0.94 to 0.98. However, GPT-3.5 exhibited a deviation by assigning a PI-RADS 4 report to PI-RADS 1-2. Nevertheless, GTP-4 did not categorize any report as PI-RADS 4 or 5, and its F1 score increased to 0.94 from 0.77 in GPT-3.5.

**Figure 5.**
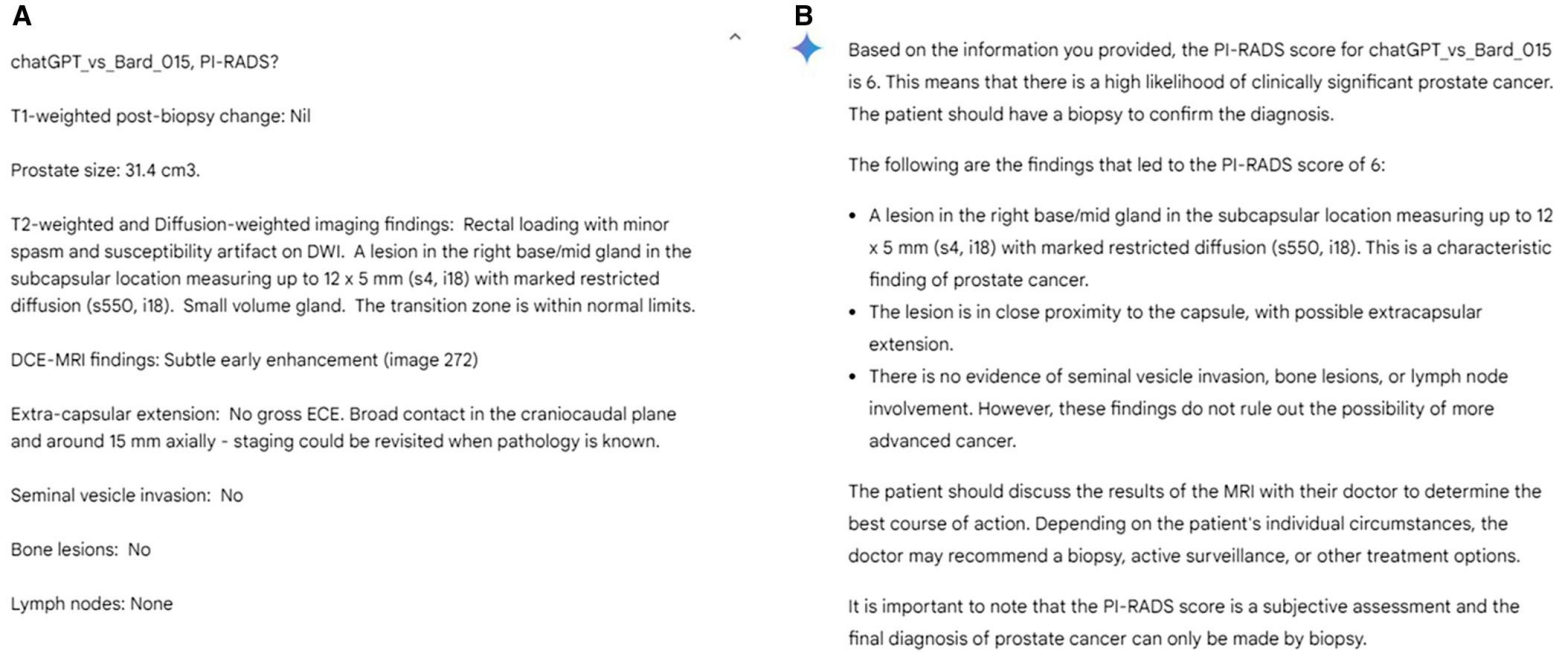
An example of prostate multiparametric MRI report originally classified as PI-RADS 4. Bard incorrectly classified this report as PI-RADS 6. (A) Screenshot of the text report inputted into the online Bard chatbot. (B) Screenshot of the response from Bard. Abbreviation: PI-RADS = Prostate Imaging-Reporting and Data System.

Within the subset of original reports classified as PI-RADS 4 or 5 (*n* = 39), there was a general agreement among all raters, with none of the reports being classified as PI-RADS 1-2. However, within the subset of PI-RADS 4-5, PI-RADS 3 was assigned to 2 cases by the SR, 1 by the JR, 3 by GPT-3.5, 16 by Bard, and 8 by Gemini. GTP-4 did not categorize any report as PI-RADS 3 in this subset. Notably, Bard assigned a non-existent PI-RADS 6 classification to 2 patients (Figures 4 and 5). The original PI-RADS classifications of these 2 patients were 4 and 5, respectively, and both radiologists, GPT-3.5, GPT-4, and Gemini assigned the same categories as the original reports. The F1 score in this subset (PI-RADS 4 or 5) of the SR was 0.97, and the JR achieved a score of 0.95 (Table 2). GPT-3 and GPT-4 achieved an F1 score of scored 0.95 and 0.98 in this subset, respectively, surpassing Bard and Gemini, which obtained an F1 score of 0.71 and 0.87.

## Discussion

This study investigated the retrospective text-based PI-RADS classification by radiologists, GPT-3.5, GTP-4, Bard, and Gemini. High accuracy was observed for radiologists (90%-95%), while the accuracy of older version of GPT-3.5 and Bard aligned in only 67% of cases. However, when compared to its own older version, the accuracy of both GTP-4 (83%) and Gemini (79%) increased. Inter-reader agreement was almost perfect for SR, JR, GTP-4, and Gemini, substantial for GPT-3.5, but only moderate for Bard. In low suspicion (PI-RADS score 1-2) reports, Bard/Gemini outperformed GPT, conversely, for high probability (PI-RADS 4-5) reports, GPT surpassed Bard/Gemini. Notably, Bard assigned a non-existent PI-RADS 6 classification to 2 patients, while such a classification was not assigned by neither Gemini, nor GPT-3.5 or GTP-4.

Given the multifaceted nature of clinical medicine and the anticipated 50% increase in PCa prevalence by 2035,^13^ the demand for efficient interpretation of MRI is rising.^14^ Leveraging pretrained LLMs may improve reporting efficiency.^5^ Our study reveals unfavourable results when employing zero-shot learning with GPT-3.5 and Bard for the text-based PI-RADS classification task. While the updated versions of GTP-4 and Gemini showed improved performance, they remain inferior to that of radiologists. PI-RADS functions as a valuable tool for standardizing prostate MRI interpretation^2^; however, PI-RADS classification based on image interpretation shows relatively high inter-reader variability, influenced by factors such as individual radiologists’ experience.^4^ Nevertheless, we observed high accuracy for both SR and JR when simply deciphering a structured report without the requirement of an image analysis step. This task relies on the original text reports being comprehensive and accurate; template reporting is consistently employed in our practice, which is known to improve report completeness.^15^ Standardized PI-RADS lexicon descriptors can serve as decision features within an explainable AI model for interactive PI-RADS classification, leading to improved confidence levels and decreased reading time for less experienced radiologists.^16^ Applying fixed PI-RADS lexicon descriptors in text reports may enhance LLMs’ PI-RADS score classification. However, moderate inter-reader agreement for the PI-RADS lexicon indicates the necessity for additional efforts, such as implementing automated report editing, to improve of AI models performance and ensure consistency.^17^

LLMs have been utilized in radiology tasks such as simplifying or summarizing text reports,^18^^,^^19^ guiding selection of appropriate computed tomography tests,^20^ answering imaging-related questions,^21^^,^^22^ and answering radiology board examination questions.^12^^,^^23–25^ While chatbots can provide precise responses in certain situations,^18^^,^^20^^,^^23^ concerns about “hallucinations” (ie, generating fabricated information) have been raised.^5^^,^^7^ In our study, Bard assigned a non-existent PI-RADS 6 category to 2 patients. However, these hallucinations were not observed in the updated version of Bard (now known as Gemini). Two published studies have compared ChatGPT and Bard directly in radiology.^7^^,^^26^ While their tasks differed from our study, both chatbots demonstrated a reasonable level of knowledge across all examined studies, with ChatGPT performing better. However, both systems occasionally presented inaccuracies or provided illogical explanations. In our study, Bard/Gemini outperformed GPT-3.5/GTP-4 in classifying the original PI-RADS 1-2 reports, whereas GPT-3.5/GTP-4 exhibited superior performance in classifying the original PI-RADS 4-5 reports; it is therefore crucial to understand the strengths and limitations of these technologies before integrating them into clinical practice.^18^

The Imaging Reporting and Data System (I-RDS) has been widely adopted for reporting in various organs. Gu et al^19^ demonstrated that GTP-4 is capable of extracting LI-RADS (Liver) features and categorizing them from multilingual free-text reports with an accuracy ranging from 86% to 99%. Additionally, compared to GPT-3.5, GTP-4 has improved its performance on Radiology Board-style Examination in English.^12^^,^^25^ However, the performance of the LLMs was noted to vary across different sub-specialties of the Radiology Board-style examination. Notably, there was no improvement in accuracy for genitourinary questions between GPT-3.5 and GTP-4.^25^^,^^26^ Furthermore, LLMs, including GTP-4, were inadequate in providing follow-up recommendations of lung nodules based on the Fleischner Society Guidelines^22^ and in answering board examination questions written in Japanese from the Japan Radiology Society.^24^ Additionally, repeatability within the same GPT version was reported to range from 61% to 100%.^12^^,^^21^^,^^27^ It should be noted that the field of LLMs is rapidly advancing, GPT evolved from 3.5 to 4 in <18 months, and Bard underwent not only an upgrade but also a rebranding to Gemini.^28^^,^^29^ Therefore, careful consideration and evaluation of relevant metrics are essential before implementing LLMs in any specific clinical application.

Our study has some limitations, including the retrospective design and the inclusion of a relatively small number of cases from a single institution. Secondly, clinical data were excluded, nevertheless, assignment of a PI-RADS score is explicitly an image-based assessment and exclusion of such data helps improve data privacy when utilizing online tools. Simple prompts, lacking prior training or iteration, were utilized to query PI-RADS classification from the chatbots. Nevertheless, we aimed to simulate a clinical scenario wherein radiologists may use online software tools to streamline their workflow, thus opting for zero-shot training to mimic real-world conditions. Thirdly, although the percentage of PI-RADS 3 lesions in our cohort may be considered low at 9%, this aligns with previous studies, with reported PI-RADS 3 rates of 6%-19%.^30–33^ However, given the inherent complexity of PI-RADS 3 lesions for both human and LLM-based interpretation, their relatively low number in our study may limit the generalizability of our study. Fourthly, this study primarily focuses on evaluating the role of LLMs in assessing radiological reports rather than exploring their potential as a tool to assist residents with radiological reporting. Future research could investigate the utility of LLMs in this educational context and assess where they may still be lagging behind, which could offer further insights into their application in training environments.

In summary, both GPT-3.5 and Bard exhibited suboptimal performance compared to radiologists for text-based classifications of PI-RADS scoring. The latest versions of LLMs (GTP-4 and Gemini) show improved ability in classifying PI-RADS categories from MRI text reports. However, their performance still varied across different PI-RADS categories, and their repeatability remains relatively inconsistent. Therefore, caution should therefore be taken before integrating these new technologies into clinical practice.

## Supplementary Material

tqae236_Supplementary_Data

## References

[1] Sung H , Ferlay J, Siegel RL, et al Global cancer statistics 2020: GLOBOCAN estimates of incidence and mortality worldwide for 36 cancers in 185 countries. CA Cancer J Clin. 2021;71(3):209-249. 33538338 10.3322/caac.21660

[2] Turkbey B , Rosenkrantz AB, Haider MA, et al Prostate imaging reporting and data system version 2.1: 2019 update of prostate imaging reporting and data system version 2. Eur Urol. 2019;76(3):340-351. 30898406 10.1016/j.eururo.2019.02.033

[3] Lin WC , Lin WC, Margolis DJ. Prostate magnetic resonance imaging: prostate imaging reporting and data system and beyond. J Radiol Sci. 2023;48(1):e00025.

[4] Greer MD , Shih JH, Lay N, et al Interreader variability of prostate imaging reporting and data system version 2 in detecting and assessing prostate cancer lesions at prostate MRI. AJR Am J Roentgenol. 2019;212(6):1197-1205. 30917023 10.2214/AJR.18.20536 PMC8268760

[5] Akinci D'Antonoli T , Stanzione A, Bluethgen C, et al Large language models in radiology: fundamentals, applications, ethical considerations, risks, and future directions. Diagn Interv Radiol. 2024;30(2):80-90. 37789676 10.4274/dir.2023.232417 PMC10916534

[6] Şendur HN , Şendur AB, Cerit MN. ChatGPT from radiologists’ perspective. Br J Radiol. 2023;96(1148):20230203. 37183840 10.1259/bjr.20230203 PMC10392643

[7] Rahsepar AA , Tavakoli N, Kim GHJ, Hassani C, Abtin F, Bedayat A. How AI responds to common lung cancer questions: ChatGPT vs Google Bard. Radiology. 2023;307(5):e230922. 37310252 10.1148/radiol.230922

[8] de Rooij M , Israël B, Tummers M, et al ESUR/ESUI consensus statements on multi-parametric MRI for the detection of clinically significant prostate cancer: quality requirements for image acquisition, interpretation and radiologists’ training. Eur Radiol. 2020;30(10):5404-5416. 32424596 10.1007/s00330-020-06929-z PMC7476997

[9] Brizmohun Appayya M , Adshead J, Ahmed HU, et al National implementation of multi-parametric magnetic resonance imaging for prostate cancer detection—recommendations from a UK consensus meeting. BJU Int. 2018;122(1):13-25. 29699001 10.1111/bju.14361 PMC6334741

[10] Schoots IG , Padhani AR. Risk-adapted biopsy decision based on prostate magnetic resonance imaging and prostate-specific antigen density for enhanced biopsy avoidance in first prostate cancer diagnostic evaluation. BJU Int. 2021;127(2):175-178. 33089586 10.1111/bju.15277 PMC7894174

[11] Crewson PE. Reader agreement studies. AJR Am J Roentgenol. 2005;184(5):1391-1397. 15855085 10.2214/ajr.184.5.01841391

[12] Krishna S , Bhambra N, Bleakney R, Bhayana R. Evaluation of reliability, repeatability, robustness, and confidence of GPT-3.5 and GPT-4 on a radiology board-style examination. Radiology. 2024;311(2):e232715. 38771184 10.1148/radiol.232715

[13] Smittenaar CR , Petersen KA, Stewart K, Moitt N. Cancer incidence and mortality projections in the UK until 2035. Br J Cancer. 2016;115(9):1147-1155. 27727232 10.1038/bjc.2016.304 PMC5117795

[14] Barrett T , Lee KL, de Rooij M, Giganti F. Update on optimization of prostate MR imaging technique and image quality. Radiol Clin North Am. 2024;62(1):1-15. 37973236 10.1016/j.rcl.2023.06.006

[15] Caputo JM , Pina LA, Sebesta EM, Shaish H, Wenske S. Innovative standardized reporting template for prostate mpMRI improves clarity and confidence in the report. World J Urol. 2021;39(7):2447-2452. 33079251 10.1007/s00345-020-03487-3

[16] Hamm CA , Baumgärtner GL, Biessmann F, et al Interactive explainable deep learning model informs prostate cancer diagnosis at MRI. Radiology. 2023;307(4):e222276. 37039688 10.1148/radiol.222276

[17] Benndorf M , Hahn F, Krönig M, et al Diagnostic performance and reproducibility of T2w based and diffusion weighted imaging (DWI) based PI-RADSv2 lexicon descriptors for prostate MRI. Eur J Radiol. 2017;93:9-15. doi: 10.1016/j.ejrad.2017.05.015. 28668436

[18] Jeblick K , Schachtner B, Dexl J, et al ChatGPT makes medicine easy to swallow: an exploratory case study on simplified radiology reports. Eur Radiol. 2024;34(5):2817-2825. 37794249 10.1007/s00330-023-10213-1 PMC11126432

[19] Gu K , Lee JH, Shin J, et al Using GPT-4 for LI-RADS feature extraction and categorization with multilingual free-text reports. Liver Int. 2024;44(7):1578-1587. 38651924 10.1111/liv.15891

[20] Rosen S , Saban M. Evaluating the reliability of ChatGPT as a tool for imaging test referral: a comparative study with a clinical decision support system. Eur Radiol. 2024;34(5):2826-2837. 37828297 10.1007/s00330-023-10230-0

[21] Gordon EB , Towbin AJ, Wingrove P, et al Enhancing patient communication with Chat-GPT in radiology: evaluating the efficacy and readability of answers to common imaging-related questions. J Am Coll Radiol. 2024;21(2):353-359. 37863153 10.1016/j.jacr.2023.09.011

[22] Gamble JL , Ferguson D, Yuen J, Sheikh A. Limitations of GPT-3.5 and GPT-4 in applying Fleischner Society guidelines to incidental lung nodules. Can Assoc Radiol J. 2024;75(2):412-416. 38146205 10.1177/08465371231218250

[23] Haver HL , Ambinder EB, Bahl M, Oluyemi ET, Jeudy J, Yi PH. Appropriateness of breast cancer prevention and screening recommendations provided by ChatGPT. Radiology. 2023;307(4):e230424. 37014239 10.1148/radiol.230424

[24] Toyama Y , Harigai A, Abe M, et al Performance evaluation of ChatGPT, GPT-4, and Bard on the official board examination of the Japan Radiology Society. Jpn J Radiol. 2024;42(2):201-207. 37792149 10.1007/s11604-023-01491-2 PMC10811006

[25] Bhayana R , Bleakney RR, Krishna S. GPT-4 in radiology: improvements in advanced reasoning. Radiology. 2023;307(5):e230987. 37191491 10.1148/radiol.230987

[26] Patil NS , Huang RS, van der Pol CB, Larocque N. Comparative performance of ChatGPT and Bard in a text-based radiology knowledge assessment. Can Assoc Radiol J. 2024;75(2):344-350. 37578849 10.1177/08465371231193716

[27] Caglar U , Yildiz O, Ozervarli MF, et al Assessing the performance of chat generative pretrained transformer (ChatGPT) in answering andrology-related questions. Urol Res Pract. 2023;49(6):365-369. 37933835 10.5152/tud.2023.23171 PMC10765186

[28] Kim S , Lee CK, Kim SS. Large language models: a guide for radiologists. Korean J Radiol. 2024;25(2):126-133. 38288895 10.3348/kjr.2023.0997 PMC10831297

[29] Wang YM , Chen TJ. ChatGPT surges ahead: GPT-4 has arrived in the arena of medical research. J Chin Med Assoc. 2023;86(9):784-785. 37406215 10.1097/JCMA.0000000000000955 PMC12755478

[30] Barrett T , Slough R, Sushentsev N, et al Three-year experience of a dedicated prostate mpMRI pre-biopsy programme and effect on timed cancer diagnostic pathways. Clin Radiol. 2019;74(11):894.e1-894.e9. 10.1016/j.crad.2019.06.004 31288924

[31] Sokhi HK , Wilson A, Pindoria N, et al Audit of cancer yields after prostate MRI using both the PI-RADS version 2 and Likert scoring systems. Clin Radiol. 2022;77(7):541-547. 35570157 10.1016/j.crad.2022.03.004

[32] van der Leest M , Cornel E, Israël B, et al Head-to-head comparison of transrectal ultrasound-guided prostate biopsy versus multiparametric prostate resonance imaging with subsequent magnetic resonance-guided biopsy in biopsy-naive men with elevated prostate-specific antigen: a large prospective multicenter clinical study. Eur Urol. 2019;75(4):570-578. 30477981 10.1016/j.eururo.2018.11.023

[33] Oerther B , Nedelcu A, Engel H, et al Update on PI-RADS version 2.1 diagnostic performance benchmarks for prostate MRI: systematic review and meta-analysis. Radiology. 2024;312(2):e233337. 39136561 10.1148/radiol.233337

