# Supplementary material for: Assessing the performance of ChatGPT and Bard/Gemini against radiologists for Prostate Imaging-Reporting and Data System classification based on prostate multiparametric MRI text reports
Source: Br J Radiol. 2024 Nov 13;98(1167):368–74. doi: 10.1093/bjr/tqae236 (PMC11840166; doi:10.1093/bjr/tqae236)
Supplement: tqae236_Supplementary_Data [file tqae236_supplementary_data.docx]

**Supplemental Tables**

| **PI-RADS** | **Radiologist 1** | **Radiologist 2** | **Radiologist 3** |
| --- | --- | --- | --- |
| **1-2** | 19 | 21 | 12 |
| **3** | 3 | 2 | 4 |
| **4** | 7 | 5 | 7 |
| **5** | 5 | 5 | 10 |

**Supplemental Table 1.** PI-RADS scores breakdown across original text reports and original report radiologists.

|  | GPT-3.5 | | GPT-4 | | Bard | | Gemini | |
| --- | --- | --- | --- | --- | --- | --- | --- | --- |
|  | Correct | Incorrect | Correct | Incorrect | Correct | Incorrect | Correct | Incorrect |
| R1 | 21 | 13 | 24 | 10 | 28 | 6 | 31 | 3 |
| R2 | 23 | 10 | 26 | 7 | 20 | 3 | 28 | 5 |
| R3 | 23 | 10 | 17 | 16 | 25 | 8 | 20 | 13 |
| *P-value* |  | *0.77* |  | *0.61* |  | *0.27* |  | *0.006* |

**Supplemental Table 2.** LLMs performance stratified by the original report radiologists. GPT-3.5 = ChatGPT-3.5, GTP-4 = ChatGPT-4o mini, R1 = Radiologist 1, R2 = Radiologist 2, R3= Radiologist 3.
